# Genes with mutation significance were highly associated with the clinical pattern of patients with breast cancer

**DOI:** 10.18632/oncotarget.21453

**Published:** 2017-10-03

**Authors:** Wan-Jun Ding, Tao Zeng, Li-Jun Wang, Hong-Bo Lei, Wei Ge, Zhi Wang

**Affiliations:** ^1^ Department of Oncology, Renmin Hospital, Wuhan University, Wuhan, Hubei 430060, P.R. China; ^2^ Department of East Campus, Renmin Hospital, Wuhan University, Wuhan, Hubei 430060, P.R. China; ^3^ Department of Breast and Thyroid Surgery, Renmin Hospital, Wuhan University, Wuhan, Hubei 430060, P.R. China

**Keywords:** breast invasive carcinoma, driver mutation, TP53, PIK3CA

## Abstract

In the United States, breast cancer is the second leading cause of cancer death in women. Over the past 20 years, breast cancer incidence and mortality rates increased rapidly in developing regions. We aimed to identify the gene mutation patterns that associated with the clinical patterns, including survival status, histo-pathological classes and so forth, of breast cancer. We retrieved 1098 cases of the clinical information, and level-3 legacy data of mRNA expression level, protein expression data and mutation files from GDC data portal. The genes with mutation significance were obtained. We studied the impacts of mutation types on the expression levels of mRNA and protein. Different statistics methods were used to calculate the correlation between the mutation types and the expression data or histo-clinical measures. There were 24 genes with mutation significance identified. The most mutated genes were selected to study the role of specific mutations played on the patients with breast cancer. One interesting finding was the missense mutations on TP53 were related with high expression levels of mRNA and protein. The missense mutations on TP53 were highly related with the morphology, race, ER status, PR status and HER2 Status, while the truncated mutations were only related with the morphology, ER status and PR status. The missense mutation on PIK3CA was highly associated with the morphology, race, ER status and PR status. The mutants with different mutants and the wild type of the most mutated genes had different impacts on the histo-clinical measures that might help personalized therapy.

## INTRODUCTION

In the United States, breast cancer is the second most commonly diagnosed cancer and the second leading cause of cancer death in female. An estimated 252,710 new cases of breast invasive carcinoma and 40,610 breast cancer deaths are expected to occur among U.S. women in 2017 [1]. Over the past 20 years, breast cancer incidence and mortality rates increased rapidly in developing regions [2]. It was estimated that about half of the new breast cancer cases and 60 % of the breast cancer deaths occurred in developing countries.

Breast cancer is considered as a highly heterogeneous disease. WHO suggested 20 major tumor types and 18 minor subtypes based on morphological features [3]. A major drawback of this classification is that most of the all breast cancers belong to two major histopathological classes. Gene expression profiling or immuno-histochemical approaches have also been used to unveil the molecular basis for heterogeneity of breast cancer like basal-like, HER2-enriched and so forth [4].

The mutational theory of cancer proposes that driver mutations provide proliferative advantage, causing outgrowth of a neoplastic clone. Multiple mutational processes, including endogenous and exogenous mutagen exposures, aberrant DNA editing and so forth, are responsible for generating these mutations [5]. Recently, DNA sequencing has enabled systematic characterization of the mutation types including single nucleotide substitutions, small insertions or deletions and copy number changes. It was the foundation of the study of driver mutations and also contributed a lot to the clinical application.

In this study, we utilized the breast invasive carcinoma datasets from TCGA to study the impact of specific mutations on genes with mutation significance (MutSig) on the histo-clinical parameters, especially the breast classification, and the mRNA or protein expression levels. We aimed to identity how the specific mutations led to the different phenotypes like the histopathological classification. In further, our analysis might help a comprehensive understanding of the origins and consequences of somatic mutations in breast cancer.

## RESULTS

### The genes with mutation significance

**Figure 1:**
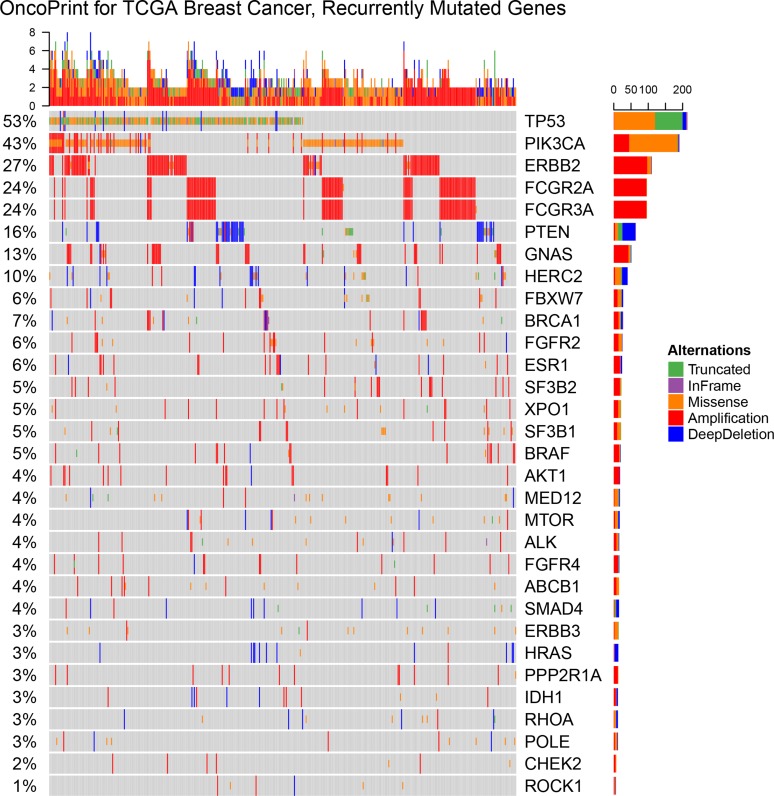
OncoPrint showing the distribution of genomic alterations in breast cancer The OncoPrint provided an overview of genomic alterations (legend) in particular genes (rows) affecting individual samples (columns). The truncated mutations, inframe deletion or insertion, and missense mutations, were shown as green, purple and orange color with the one-thirds height. And copy number variations were annotated with the full height that amplification was red and the copy number loss was blue. The same color palettes of alterationswere used in the following graphs as well.

The somatic mutations and copy number variation of the patients with at least one alterations in the 24 MugSig genes were shown in the Oncoprints (Figure 1). Individual genes were represented as rows, and individual cases were represented as columns. The SNPs, including truncated mutations, inframe deletion or insertion and missense mutations, were shown as green, purple and orange color with the one-thirds height, respectively. And the rectangle with full height showed the copy number variation: the red was amplification and the blue was the copy number loss.

### Theimpact of mutations on the mRNA and protein expression level

The top MugSig genes, including TP53, PIK3CA, ERBB2 and PTEN were selected to study the role of specific mutations played on the patients with breast cancer. The reason that FCGR2A and FCGR3A were excluded even though they had even more alteration cases than PTEN was that the genomic position of FCGR2A and FCRG3A were quite close and their alteration patterns were almost the same. It was reported that the CNV occurred in the locus where FCGR2A and FCRG3A were located. However, the amplification of FCGR2A and FCRG3A should not always occur simultaneously. For example, some population did have amplification of FCGR3A but not FCGR2A [6]. As a result, we tend to consider the high correlation of amplification of these two genes in this case would be artificial and they were excluded in the following analysis. The counts of cases in each group were listed in Table 1.

**Table 1:** The count of cases in each mutation types of TP53, PIK3CA, ERBB2 and PTEN

	Wild type	Missense	In-frame	Truncated	Amplified	Deleted	Mixed
TP53	631	146	5	95	0	11	4
PIK3CA	608	228	5	2	30	1	18
ERBB2	768	12	0	0	109	1	2
PTEN	811	14	1	15	4	47	0

**Figure 2:**
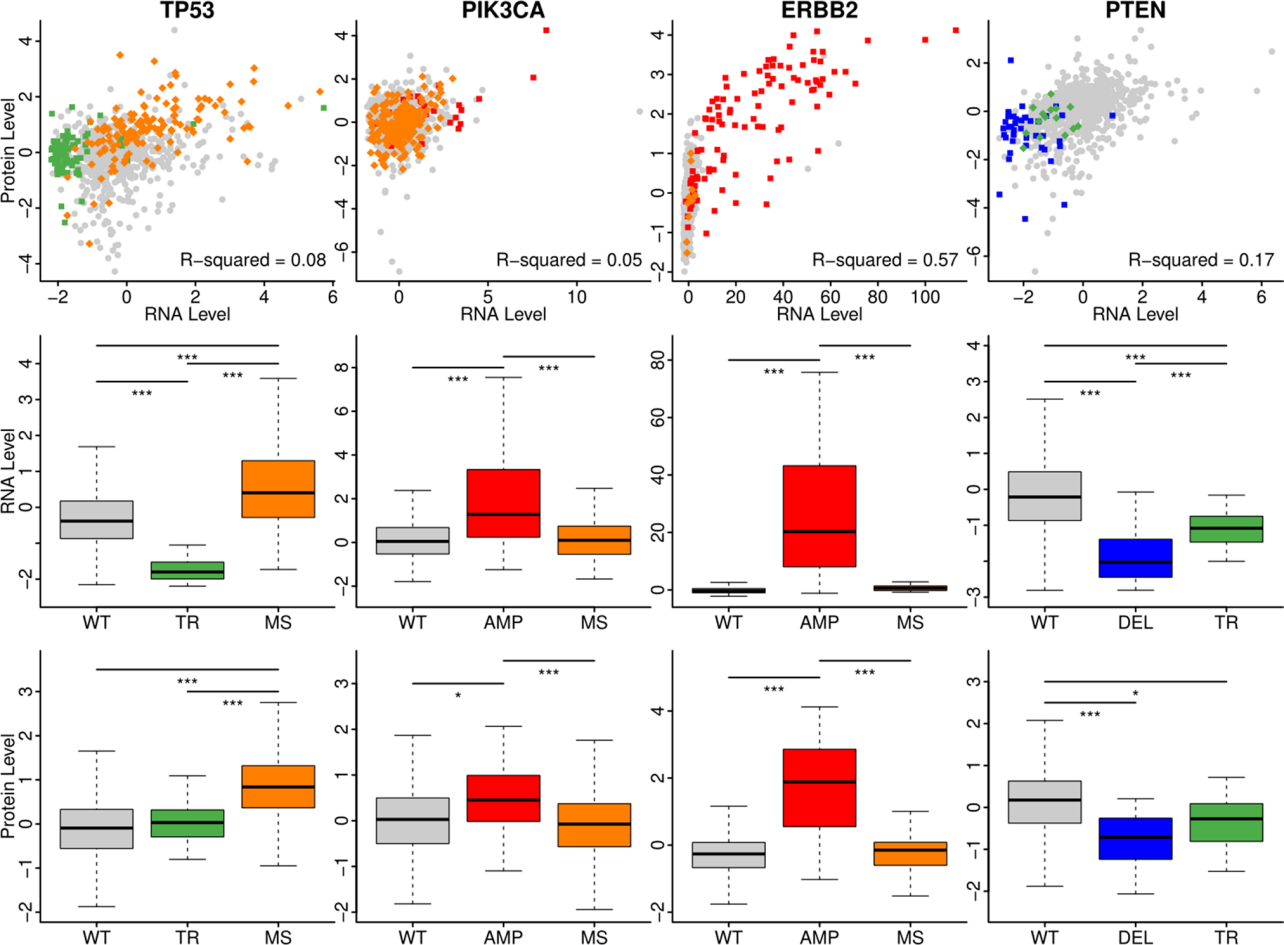
The impact of mutations on the mRNA and protein expression level The mRNA levels and protein levels were plotted as scatter with the various color annotating the mutation types. The boxplot showed the different levels of mRNA or proteins in different groups. WT: wild-type; TR: truncated mutations; MS: missense mutations; AMP: CNV amplification; DEL: CNV deletion. The color palettes were the same as Figure 1. The stars annotated the scale of *p*-value. ^*^: 0.01; ^**^: 0.005; ^***^: 0.001.

We selected the top 2 mutation types for each gene, and their influence on their mRNA expression levels and protein expression levels were plotted in (Figure 2). First, the correlation between mRNA expression levels and protein expression levels was high in ERBB2 using Pearson correlation, while the correlation was low for the other three genes. Using the Kruskal–Wallis test, the mRNA expressions and protein expression levels were all significant among the wild-type and other mutation types of the four selected genes. The segmentation duplication of PIK3CA and ERBB2 led to high expression of both mRNA and protein, but the missense did not have influence. Both truncated mutations and copy number deletion of PTEN caused the low expression levels of mRNA and protein. One interesting finding was about TP53. The truncated mutations in TP53 led to low mRNA expression but had the similar protein expression as the wild-type. On the other hand, the missense mutations were related with high expression level of mRNA and protein. From the scatter plot, it was clear that patients with missense mutations on TP53 had totally different pattern compared with wild-type or with truncated mutations.

**Figure 3:**
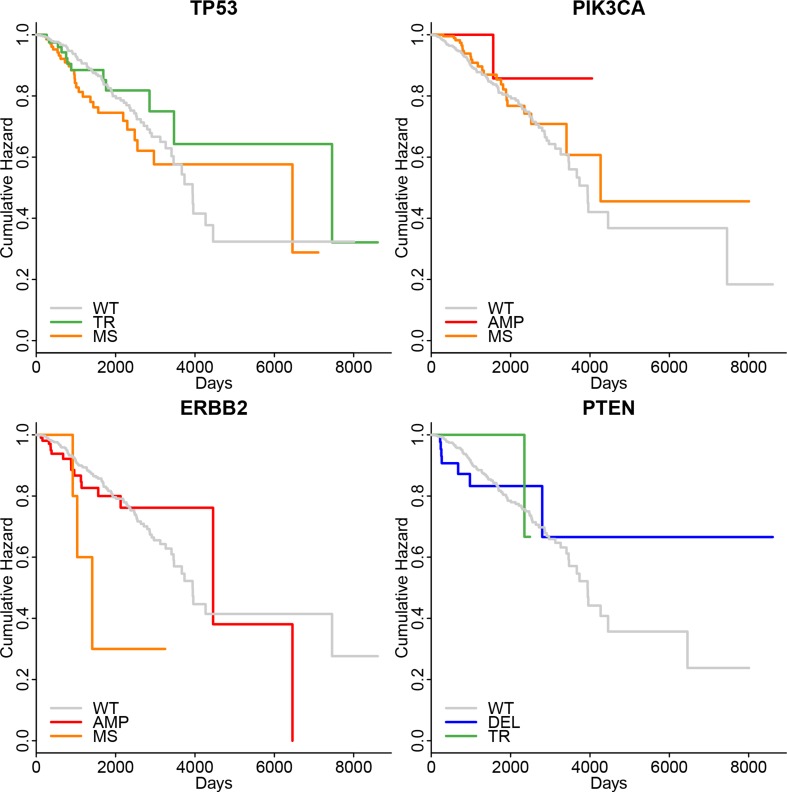
The Meier-Kaplan curve of survival status of patients in different mutation groups of selected genes None of them had the *p*-value lower than 0.05. The abbreviations were the same as Figure 2.

When we investigated whether the survival status were correlated with the specific mutations (Figure 3), we were discouraged that there were no signification difference of survival time between patients with wild type and patients with specific mutations. However, we were still curious how the mutations influences other histo-clinical information.

### The histo-clinical measures with TP53 and PIK3CA

**Figure 4:**
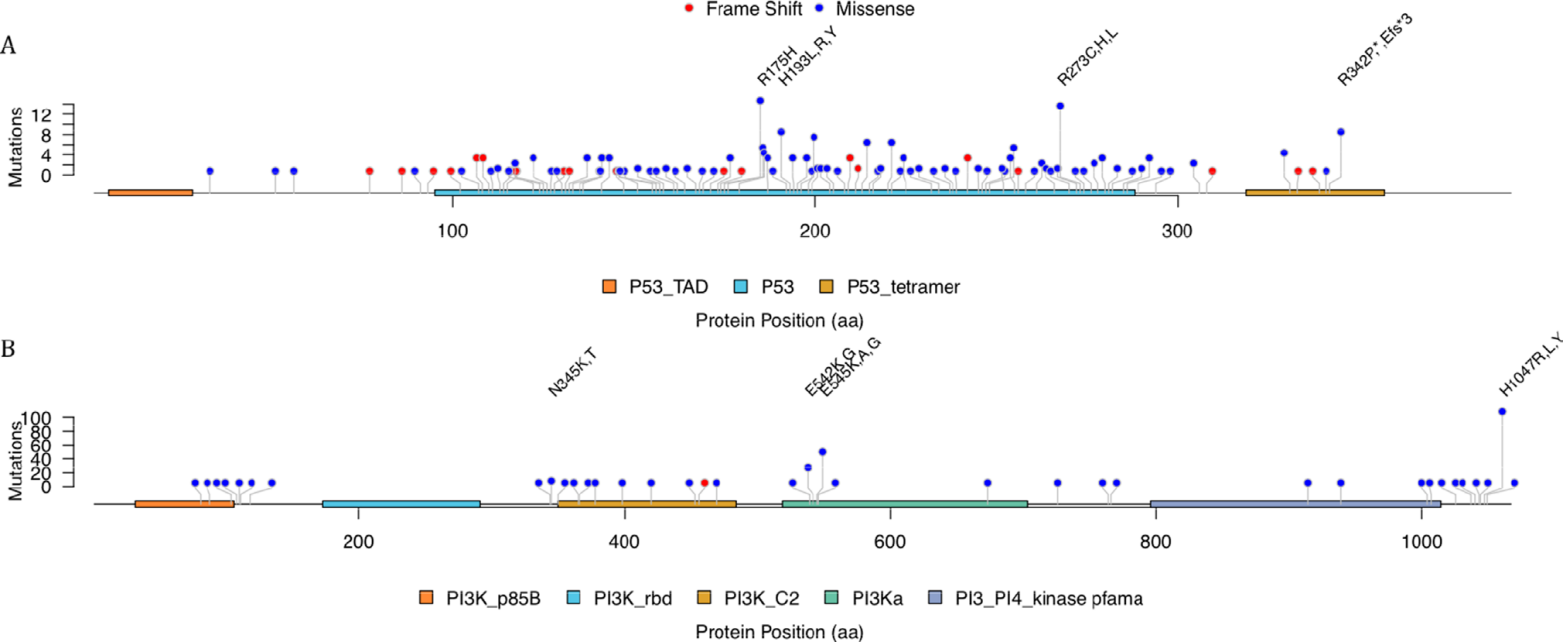
The mutation distribution over the TP53 and PIK3CA protein

Considering the relatively fewer cases with specific mutations, our foci were mainly put on missense mutations of TP53 and PIK3CA. The detailed truncated and missense mutations of TP53 and missense mutations of PIK3CA were plotted as lollipop plot (Figure 4). Though TP53 had much more mutations than PIK3CA and the exon length were shorter, the missense mutations on TP53 were relatively evenly distributed. On the other hand, PIK3CA were concentrated on several loci. There were 55 cases had missense mutation on the position of 1047 amino acid and the mutations were mainly H1047R. Actually, the missense mutation on this site were annotated as oncogenic mutations with gain of function in Precision Oncology Knowledge Base (OncoKB, http://oncokb.org/).

The correlation between specific mutations on TP53 or PIK3CA and the histo-clinical parameters were analyzed (Table 2). The missense mutations on TP53 were highly related with the morphology, race, ER status, PR status and HER2 Status, while the truncated mutations were highly related with the morphology, ER status and PR status. The missense mutation on PIK3CA were highly associated with the morphology, race, ER status and PR status. When we limited the missense mutation to the mutations on 1047 aa, the associated histo-clinical estimates were only race and PR status and the *p*-value for age at diagnosis was 0.027.

**Table 2:** The *p* value of the specific mutations on histo-clinical measures when compared with the wild-type

	TP53		PIK3CA	
	Missense	Truncated	Missense	H1047R
survival	1.40E-01	4.78E-01	2.86E-01	8.82E-01
age at diagnosis	3.34E-01	1.42E-01	2.01E-01	2.74E-02
tumor stage	8.25E-01	2.03E-01	3.92E-01	4.09E-01
morphology	1.31E-07	7.50E-07	8.19E-03	4.24E-01
race	4.61E-04	2.77E-01	9.18E-06	3.85E-03
ER status	7.32E-16	2.20E-16	4.09E-07	1.99E-01
PR status	2.32E-10	2.72E-13	1.74E-09	9.06E-05
HER2 Status	2.82E-04	9.16E-02	1.24E-01	6.08E-01
Node Coded	6.79E-01	3.94E-01	9.29E-01	7.16E-01
Metastasis	7.11E-01	1.00E+00	5.67E-01	4.17E-01
T stage	5.41E-01	1.23E-02	1.16E-01	9.72E-02

## DISCUSSION

### The missense and truncated mutations on TP53

Among 892 cases with sequencing data, 631 patients had wild-type TP53 gene even though the expression levels of mRNA or protein might aberrant or its function might be affected by the micro-environment. In other words, about 29.3% of patients with breast invasive carcinoma had mutations or copy number alterations on genes encoding TP53. Among these patients, most had missense mutations which accounted for around 55.9% and about 36.4% had truncated mutations which included nonsense mutations and frameshift deletion or insertion (Table 1).

One interesting finding is that the missense mutations on TP53 led to high expression levels of both the mRNA and protein of TP53, while truncated mutations only led to low mRNA expression levels. Such results suggested that these missense mutations had gain-of-function. In fact, TP53 had quite unique mutation patterns when compared with other tumor suppressor genes. Most of tumor suppressors were frequently inactivated by frame shift or nonsense mutations leading to either production of truncated proteins or complete elimination of the corresponding gene products just as other MugSig genes found in this study, while most of the mutations within the TP53 gene were missense mutations, resulting in the expression of full-length mutant p53 proteins. Moreover, the mutated p53 proteins became highly expressed in human cancers, which suggested the existence of a strong selection for mutant p53 overexpression in breast cancer. Functionally, TP53 missense mutations resulted in loss of the wild-type tumor suppressor activities, and also may suppress the function of the wild-type TP53 allele via a dominant-negative mechanism. It also was reported that TP53 missense might gain of oncogenic function [7].

The mutations of TP53 showed several hotspots even though most of mutations only appeared in one case. Such hotspot contains R175H, R273H and H193L,R,Y. It was shown that both R175H and R273H were oncogenic mutations. R175H mutation was a structural mutation which led to an unfolded DNA-binding domain while R273H were contact mutation by substituting the interacted residues. But H193L,R,Y seemed to be uncommon, and only H193R was annotated as likely to be oncogenic and might be loss-of-function according to OncoKB. It was also reported that there was mutant-specific p53 reactivator, like NSC319726. Yu, Vazquez [8] showed that this drug specifically targeted the p53 hotspot mutant R175H. It can restore wild-type conformation and trigger R175H-dependent cell death by apoptosis [9]. Further drug discovery should be put on other frequent p53 mutants, such as other hotspot we found like R273H and H193L,R,Y.

### Theimpact of TP53 mutations onhisto-clinical measures

After analyzing the correlation with TP53 mutations and the histo-clinical parameters, the results showed that the missense mutations on TP53 were highly related with the morphology, race, ER status, PR status and HER2 Status, while the truncated mutations were highly related with the morphology, ER status and PR status.

Among cases with wild-type TP53, over 65% had morphology with a code of 8500/3, which meant as infiltrating duct adenocarcinoma without other specific and about 23% had a code of 8520/3 as lobular carcinoma without other specific (Table 3). However, in the patients with mutated TP53, the ratio of 8520/3 were lower than 5%. There were two studies which showed that out of total 48 breast cancers with TP53 mutations were all tumors of the ductal type but none of them were lobular type [10, 11]. Their results supported our findings that TP53 mutations predispose to duct adenocarcinoma but not to lobular carcinoma.

**Table 3:** The association of selected clinical parameters and TP53 mutations

Clinical Information		WT	MS	TR
Morphology	8500	408	124	84
	8520	144	6	2
	8522	18	3	2
	8523	13	1	2
	other	42	11	4
Race	Asian	29	21	8
	Black or African American	94	20	15
	White	458	97	66
ET	Negative	47	55	50
	Positive	375	63	32
PR	Negative	98	65	53
	Positive	322	54	27
HER2	Negative	372	88	63
	Positive	47	30	14

On the meantime, missense and truncated mutations also performed differently according to the ER, PR and HER2 status. Our findings were in agreement with previous comparisons of the breast tumor intrinsic subtypes and TP53 mutation status. It was reported that about 44% of basal-like tumors and 43% of HER2+/ER− subtype tumors contained TP53 mutations, while lower than 23% of luminal were mutation-positive [12].

Though the tumor suppressor role of TP53 were quite clear, it was still unclear why the mutation patterns of TP53 were different from the different morphology or the presentation of antibody, whether the mutations had impacts on them and how the specific mutations might contribute.

### The missense mutation of PIK3CA and H1047R

PIK3CA had obvious mutation hotspots and the most significant one was on H1047R. And the hotspots also included E545K. In fact PIK3CA were one of the most mutated genes in various cancers like breast, colon, glioblastomas and so forth. It was reported that about eighty percent of the mutations were H1047R or a cluster of 542 aa, 545 aa and 546 aa [13].

Meyer, Brinkhaus [14] found that expression of the PIK3CA mutant with H1047R was sufficient to induce tumor formation in transgenic mice. Also it was suggest that the mutant H1047R prevented cell death by increased PI3K/AKT pathway activation [15]. Gkeka, Evangelidis [16] presented a model of the over-activation mechanism of the H1047R based on structural and dynamic differences with the wild-type. All these studied illustrated the extreme importance of PIK3CA mutant H1047R.

The relationship between PIK3CA mutations, especially H1047R, and various histo-clinical parameters were identified using various statistical methods. Most of our results showed concordance with the previous studies. PIK3CA mutations had been found at similar frequencies in breast ductal and lobular carcinoma [17], though we found there were a small difference based on TCGA breast cancer dataset (Table 4). We had exact the same finding when it comes to the PR, ER and HER2 receptor. Pang, Cheng [18] carried out a meta-analysis involving 26 studies and they indicated the significant association between PIK3CA mutations and ER and PR expressions. In terms of HER2 status, most of studies demonstrated no association of PIK3CA with HER2 status [19].

**Table 4:** The association of selected clinical parameters and PIK3CA mutations

Clinical Information		WT	MS	H1037R
Morphology	8500	434	149	69
	8520	99	53	19
	8522	12	11	5
	8523	11	4	1
	other	47	9	7
Race	Asian	34	18	12
	Black or African American	111	14	8
	White	416	181	73
ER	Negative	117	16	11
	Positive	304	154	70
PR	Negative	171	27	15
	Positive	247	143	66
HER2	Negative	343	155	68
	Positive	65	19	10

Even though PIK3CA was the most mutated genes in multiple cancers, there was few studies reporting the drug sensitivity or outcomes when the mutation was presented. As a result, more efforts were needed to tell how and whether specific mutations in MutSig genes affected the disease or the therapy.

## MATERIALS AND METHODS

### Breast cancer provisional data from GDC

There were totally 1098 cases in the provisional breast invasive carcinoma datasets from GDC data portal. The clinical information and classification of subtypes were retrieved using TCGAbiolinks on Jun 19, 2017 [20]. The level-3 legacy data of transcriptome profiling from Illumina RNA-Seq platform, processed mutation files from Exome sequencing and protein expression data from MDA RPPA (MD Anderson Reverse Phase Protein Array) Core were also downloaded using GDCquery [20].

### Genes with mutation significance

The genes with mutation significance were calculated using the number of mutations and the number of covered bases for each gene. The genes with mutations or copy number alterations in at least of 1% patients were extracted.

The single-nucleotide polymorphism (SNP) and copy number variation of these 24 MutSig genes were visualized using OncoPrint [21, 22] and plotted with R package ComplexHeatmap [23]. In order to visualize the mutation positions and patterns, lollipop plot was created using R package trackViewer [24], where the x-axis was the position of amino acid and the y-axis was the occurrence of mutations on the given position and the dominant types of mutations were annotated by different colors.

### The role of mutations on the RNA and protein expression level

The patients were separated into three groups according to their mutation types of TP53, PIK3CA, ERBB2 and PTEN. We selected these four genes because they had the lowest *p*-value that they were significantly mutated in patients when compared with normal samples except for FCGR2A and FCGR3A. The reason that FCGR2A and FCGR3A were excluded was that the genomic position of FCGR2A and FCRG3A were quite close and their alteration patterns were almost the same. Such similar pattern might be artificial by experimental strategy but not the genuine. We selected the top 2 mutation types for each gene, and studied their impacts on the expression levels of mRNA and protein.

The Kruskal-Wallis rank sum test, which was a non-parametric method for testing whether the location parameters of the distribution of cases were the same in each group without assumption of normal distribution, was utilized in order to figure out whether there were differences of mRNA or protein expression levels on patients with wild-type MugSig genes or with specific mutations [25]. And the scatter plot using mRNA expression data as x-axis and protein expression levels as y-axis were created and the mutation status were shown using different color. Wild-type, missense mutations, truncated mutations, copy number amplifications and copy number deletions were annotated by grey, orange, green, red and blue, respectively.

### The relation between mutations with histo-clinical evaluation

We would like to identify whether specific type of mutations have different influence on the RNA expression level, protein expression levels, protein functions, and the histo-clinical features. Different statistical methods were used including two-sample Wilcoxon tests for age at diagnosis [25], log-rank test for survival status [26] and Fisher's exact test for all other histo-clinical parameters [27]. In order to visualize the survival status, the Kaplan-Meier curves were plotted.

## CONCLUSIONS

We obtained the genes, which were highly mutated in patients with breast cancer, and most of these genes have been reported to be associated with breast cancer. Furthermore, we studied the impact of different mutation on TP53 and PIK3CA on the mRNA or protein expression, and other clinical information. We found that missense mutations on TP53 were related with high expression levels of mRNA and protein. The mutations on TP53 and PIK3CA were highly related with the morphology, race, ER status, PR status and HER2 status. These results suggested that the different mutations on specific genes might have distinct impact on the phenotypes, which was able to help diagnosis and personal treatment.
